# Prevalence and determinants of use of complementary and alternative medicine by hypertensive patients attending primary health care facilities in Kinshasa, Democratic Republic of the Congo: a cross-sectional study

**DOI:** 10.1186/s12906-017-1722-3

**Published:** 2017-04-08

**Authors:** Aimée M. Lulebo, Mala A. Mapatano, Paulin B. Mutombo, Eric M. Mafuta, Gédéon Samba, Yves Coppieters

**Affiliations:** 1 Department of Epidemiology and Biostatistics, Kinshasa School of Public Health, Faculty of Medicine, University of Kinshasa, PO Box11850, Kinshasa1, Democratic Republic of the Congo; 2 Programme National de lutte contre les maladies tropicales négligées, Kinshasa, Democratic Republic of the Congo; 3 School of Public Health, Université Libre de Bruxelles (ULB), Bruxelles, Belgium

**Keywords:** Hypertension, Complementary and alternative medicine, Medication adherence, DRC

## Abstract

**Background:**

In the Democratic Republic of the Congo the control of hypertension is poor, characterized by an increasing number of reported cases of hypertension related complications. Poor control of hypertension is associated with non-adherence to antihypertensive medication. It is well established that the use of complementary and alternative medicine is one of the main factors of non-adherence to antihypertensive medication. The aim of this study is to determine the prevalence and factors associated with the use of complementary and alternative medicine.

**Methods:**

A cross-sectional study was carried out at the Kinshasa Primary Health-care (KPHC) facilities network in November 2014. A structured interview questionnaire was administrated to a total of 280hypertensive patients. Complementary and alternative medicine were defined according to the National Institute of Health classification as a group of diverse medical and healthcare systems, practices, and products that are not presently considered to be part of conventional medicine. Data were summarized using proportion and mean (with standard deviation). The student’s *t* test and χ^2^ test were used respectively for mean and proportion comparison. Logistic regression analysis identified determinants of the use of complementary and alternative medicine.

**Results:**

The prevalence of use of complementary and alternative medicine was 26.1% (95% CI: 20.7% - 31.8%).Determinants of use of complementary and alternative medicine included misperception about hypertension curability (OR = 2.1; 95%CI: 1.1-3.7) and experience of medication side effects (OR = 2.9; 95%CI: 1.7-5.1).

**Conclusion:**

The use of CAM for hypertensive patients is a major problem; antihypertensives with fewer side effects must be emphasized. Religious leaders must become involved in the communication for behavioral change activities to improve the quality of life for hypertensive patients.

**Electronic supplementary material:**

The online version of this article (doi:10.1186/s12906-017-1722-3) contains supplementary material, which is available to authorized users.

## Background

African countries are currently undergoing one of the most rapid epidemiological transitions. These countries have been characterized for a long time by incidence of infectious diseases, demographic and nutritional transitions contributing to a growing prevalence of non-communicable diseases (NCDs) such as hypertension (HTN) [1, 2]. The number people with hypertension increased from 600 million in 1980 to 1 billion in 2008 [3]. HTN prevalence is higher in Africa (46%) compared to America (35%) [4]. In the Democratic Republic of the Congo (DRC), according to the World Health Organization (WHO), the prevalence of HTN was 32.1% in men and 31.5% in women in 2014 [5].

African countries are faced with the double burden of an increasing prevalence of HTN and other NCDs whilst simultaneously experiencing a high prevalence of infectious diseases that their Health Systems (HS) are unprepared to deal with [6, 7]. The African population, accustomed to acute symptomatic infectious disease, is probably struggling to adapt to chronic diseases which are most of the time asymptomatic and requiring lifelong treatment.

Previous studies show that Africans were less adherent than Caucasians [8] to NCD treatment leading to poor control and higher incidence of complications. Uncontrolled HTN is one of the main cardiovascular risk factors (CVRF); it is one of the leading causes of premature death as it is associated with9.4 million deaths worldwide every year [9].

Non-adherence to antihypertensive medication (NAM) is an important factor of uncontrolled HTN [10]. In the DRC, the proportion of HTN control is very low with a high incidence of HTN-related complications including stroke, chronic kidney disease (CKD) [11–13]. A study carried out in Kinshasa in 2013 showed that only 15.6% of patients attending the primary health care facilities in Kinshasa had their blood pressure under control. Uncontrolled blood pressure was statistically associated with NAM. The same study also found that 20.5% of patients who took non-prescribed medication were twice more likely to be non-adherent to treatment than other patients [14]. This led us to believe that these hypertensive patients probably use CAM.

Adherence to treatment is mainly influenced by the perception of a patient towards the effectiveness of treatment and the quality of health care (its availability and affordability, and the relationship between patient and provider).In the event where care provided by a conventional health system is dissatisfactory, patients seem more likely to use alternative care currently known as complementary and alternative medicine (CAM) [15]. CAM is defined as: ‘a group of diverse medical and healthcare systems, practices, and products that are not presently considered to be part of conventional medicine’ [16].

This study was conducted to measure the prevalence of CAM and to identify its determinants. By understanding the predictors of CAM use, it could help to make place for interventions for improving treatment adherence and control of HTN.

## Methods

### Study design

In November 2014, a cross-sectional study was carried out at the Kinshasa Primary Health-care (KPHC) facilities network. This network consists of fifty-one facilities. It allows for follow-up of patients with NCDs such as hypertension and diabetes [14].

### Study population

The study population included in the study were hypertensive patients >18 years old attending the KPHC facilities. Hypertensive pregnant women were excluded.

### Sampling

Fifteen out of fifty-one health facilities organized patients’ visits during the study period and were thus all included in the study. All patients present at the facility at the time of the visit and who met the inclusion criteria were enrolled. The sample size was computed using the following formula:

$$ n\ge \frac{{Z_{\alpha}}^2. p. q}{d^2} $$

Where - **p** represents the proportion of hypertensive patients using CAM (we assumed a *p* = 19.5%) [17];**q**(1-p);**z** the value of the standard normal distribution coefficient corresponding to a significance level of alpha of 0.05 (1.96) and; **d** the precision degree that we assumed to 5% too. The minimal size computed was 246 patients.

### Data collection and study variables

Face-to-face interviews using structured questionnaires were performed by six trained data collectors. The questionnaires were previously pretested with twenty-four patients who did not included in the analysis. The questionnaires were also translated into local language (Lingala) and back-translated in French before data collection. The following variables were collected:

- Demographic and social characteristics of patients (sex, age, educational level, religious affiliation, marital status, occupation);
- Clinical characteristics of patients (duration of HTN, co-morbidity, experience of medication side effects, treatment adherence);
- Patient-related variables (knowledge of HTN risk factors and complications, knowledge of treatment benefit, perception of hypertension gravity, treatment effectiveness and HTN curability);
- Healthcare system variables (patient-provider relationship, waiting time, affordability and availability of healthcare); and
- CAM use description (use of CAM, CAM patterns, reasons for CAM use, information source about CAM).

To measure the use of CAM, the following questions were asked of the patient: “Do you currently use any method or substance other than those prescribed by your healthcare provider? If yes, what do you use?” A CAM user was defined as a patient who declared using one or more of the CAM categories in line with the definition provided by the National Institute of Health (NIH). NIH classifies CAM in 5 categories:

1. Alternative medical systems (e.g. traditional oriental medicine, acupuncture, Ayurveda, naturotherapy…).
2. Mind body intervention (meditation, hypnosis, dance, art, music-therapy, spiritual healing and prayer).
3. Biologically based therapies (herbal medicine and dietary supplements, special diets and orthomolecular medicine).
4. Manipulative and body based methods (chiropractic, massage…).
5. Energy therapies [16].

Comorbidity was defined in this study as HTN associated with Diabetes Mellitus (DM) or organ damage targets (heart disease, strokes, or CKD). Self-reported medication adherence was measured using the Morisky Medication Adherence Scale (MMAS), which is a validated scale with a good internal consistency [18, 19]. Patient responses of “yes” or “no” to the four questions were categorized into three groups, namely high, medium and low adherence, as summarized in Table 1. Thereafter, adherence was dichotomized in order to facilitate statistical analysis. Low and medium adherence was merged and classified as non-adherent and patients with high adherence were classified as adherent, as we have discussed previously [14].

**Table 1:** Morisky scale

High adherence, if the sum = 0; medium adherence, if the sum is comprised between 1 and 2 and low adherence if the sum is comprised between 3 and 4.		
Have you ever forgotten to take your BP medicine?	0. No	1. Yes
Are you sometimes careless in regard to your medicine?	0. No	1. Yes
Do you skip your medicine when you are feeling well?	0. No	1. Yes
When you feel bad due to the medicine, do you skip it?	0. No	1. Yes

Source: Morisky et al. (1986). Concurrent and predictive validity of a self-reported measure of medication adherence.Med Care.1986; 24: 67-74

Availability of healthcare facility (HCF) was determined by whether a HCF was available within a 5kilometerradius or 30 min walking distance from the patient. Affordability was defined by reference to the healthcare cost perception.

### Statistical analysis

Stata version 12.0was used for statistical analysis. Descriptive statistics were used to summarize the characteristics of the study population. Continuous variables were reported using mean with standard deviation. Means for age and BP of CAM users and non-CAM users were compared using the Student’s *t* test. Categorical variables were reported as a frequency and percentage and groups were compared using the χ^2^ test. The forward stepwise logistic regression helped to identify independent predictors of CAM usage. All variables associated with CAM usage in the bivariate analysis were included in the final model. The odds ratio (OR) with a corresponding 95% confidence interval was reported to quantify the strength of association. Significance was set at *p*-value of less than 0.05.

## Results

### Assessment of CAM use

A total of 280 patients were interviewed. Tables 2 and 3 describe the CAM practices. Approximately a quarter of patients declared having used CAM, indicated by a prevalence of 26.1% (95%CI: 20.7% - 31.8%). Herbal medicines (42.5%) and prayer (35.6%) were the patterns of CAM use. The reasons mentioned by HTN patients for CAM use were effectiveness (34.2%) and lower cost (28.8%). Friends (47.9%) and family members (35.6%) were the sources of information on CAM.

**Table 2:** Frequency of CAM use

Variable *n* = 280	*N* (%)
Use of CAM	
Yes	73 (26.1)
No	207 (73.9)
Total	280 (100.0)

**Table 3:** Description of CAM use

Variables *n* = 73	*N* (%)
Patterns of CAM	
Traditional medicine (self-medication/traditional healers)	31 (42.5)
Prayer	26 (35.6)
Chinese medicine	13 (17.8)
Sources of information	
Friends	35 (47.9)
Family members	26 (35.6)
Professional area	9 (12.3)
Media (radio, TV…)	8 (11.0)
Reasons of CAM usage	
Effectiveness of CAM	25 (34.2)
The low cost of CAM	21 (28.8)
Holistic care	19 (26.0)
Cultural reasons	11 (15.1)
CAM has little or no side effects	9 (12.3)

### Demographic, social and clinical characteristics of participants

Tables 4 and 5 summarize the socio-demographic and clinical characteristics of patients respectively. The mean age of participants was 60.1 ± 10.6 years and 68.6%were women; almost half (52.5%) were married or cohabiting and 43.2% were Catholics; almost half (48.9%) were unemployed and slightly more than half (51.1%) had completed at least secondary school. No statistical difference of socio-demographic characteristics was observed between CAM users and CAM non-users.

**Table 4:** Demographic and social characteristics of hypertensive patients using or not using CAM

	Overall *n* = 280 (% or SD)	CAM users *n* = 73 (% or SD)	CAM non-users *n* = 207 (% or SD)	*p*
Mean age, years (SD)	60.1 ± 10.6	58.7 ± 9.8	60.6 ± 10.8	0.190
Sex				
Female	192 (68.6)	50 (68.5)	142(68.6)	0.987
Male	88 (31.4)	23 (31.5)	65 (31.4)	
Religion				
Catholic	121(43.2)	27 (37.0)	94 (45.4)	0.287
Pentecostal	57 (20.4)	20 (27.4)	37 (17.9)	
Protestant	48 (17.1)	9 (12.3)	39 (18.8)	
Kimbanguist	22 (7.9)	7 (9.6)	15 (7.3)	
Jehovah’s witnesses	13 (4.6)	3 (4.1)	10 (4.8)	
Others	19 (6.8)	7 (9.6)	12 (5.8)	
Marital status				
Married/cohabiting	147(52.5)	41 (56.2)	106(51.2)	0.466
Single/separated/divorced/widowed	133(47.5)	32 (43.8)	101(48.8)	
Occupation				
No	137 (48.9)	41 (56.2)	96 (46.4)	0.150
Yes	143 (51.1)	32 (43.8)	111 (53.6)	
Educational level				
Primary	85 (30.4)	18 (24.7)	67 (32.4)	0.213
Secondary	144 (51.4)	44 (60.3)	100 (48.3)	
Post-secondary	51 (18.2)	11 (15.0)	40 (19.3)

**Table 5:** Clinical characteristics of hypertensive patients using or not using CAM

	Overall *n* = 280 (% or SD)	CAM users *n* = 73 (% or SD)	CAM non-users *n* = 207 (% or SD)	*p*
Duration of HTN				
≥ 5 years	89 (31.8)	23 (31.5)	66 (31.9)	0,953
< 5 years	191(68.2)	50 (68.5)	141(68.1)	
Co morbidity				
Yes	158 (56.4)	37 (50.7)	121(58.5)	0,249
No	122 (43.6)	36 (49.3)	86 (41.5)	
Experiencing side effects				
Yes	110 (39.3)	44 (60.3)	66 (31.9)	0,000^a^
No	170 (60.7)	29 (39.7)	141(68.1)	
Treatment adherent (*n* = 265)				
No	198 (74.7)	76 (71.7)	122 (76.7)	0.356

^a^Statistically significant

Moreover two-thirds (68.3%) of participants suffered from HTN for less than 5 years and 56.4% experienced co morbidities, mainly diabetes. Almost one-third (39.3%) of patients experienced side effects from medication; the CAM users declared experiencing more antihypertensive side effects than CAM non-users (60.3% vs 31.9%) (*p* = 0,000). The use of the Morisky Scale showed that 74.7% of participants (95% CI: 69.4%-79.9%) were non-adherent to their medication (NAM). However, NAM was not associated with the CAM use.

### Patients’ knowledge and perception about HTN

Table 6 summarizes the knowledge and perception of participants on HTN and its treatment. The majority of patients in both groups were unable to mention at least three risk factors of HTN (94.3%) and HTN complications (94.3%). Only 13.9% of the patients had good knowledge of the benefit of antihypertensive treatment. A lack of knowledge of the benefit of antihypertensive treatment was associated with CAM use (*p* = 0.022). Most patients (82.4%) knew that HTN was a serious health problem. Close on two-thirds (61.4%) of patients thought of HTN as curable. This misperception of HTN curability was statistically associated with CAM use (*p* = 0.045).

**Table 6:** Use of CAM,knowledge and perception of HTN

	Overall *n* = 280 (% or SD)	CAM users *n* = 73 (% or SD)	CAM non-users *n* = 207 (% or SD)	*p*
Knowledge of HTN risk factors				
Have cited a least three	16 (5.7)	3 (4.1)	13 (6.3)	0.492
Have cited less than three	264 (94.3)	70 (95.9)	194 (93.7)	
Knowledge of HTN complications				
Have cited a least three	16 (5.7)	1 (1.4)	15 (7.2)	0.063
Have cited less than three	264 (94.3)	72 (98.6)	192 (92.8)	
Knowledge of treatment benefits				
Have cited less than 3	241 (86.1%)	57 (78.1%)	184 (88.9%)	0,022^a^
Have cited a least three	39 (13.9%)	16 (21.9%)	23 (11.1%)	
Perception of HTN gravity				
HTN is not a serious health condition	49 (17.5%)	14 (19.2%)	35 (16.9%)	0,673
HTN is a serious health condition	231(82.5%)	59 (80.8%)	172 (83.1%)	
Perception HTN curability				
HTN is curable	172 (61.4%)	52 (71.2%)	120 (58.0%)	0,045^a^
HTN is not curable	108 (38.6)	21 (28.8%)	87 (42.0%)	

^a^Statistically significant

### Healthcare system factors

Table 7 summarizes the health team and system variables. The study shows that the majority of patients declared that healthcare facilities were near their households (87.9%). Appreciation of the relationship with health care providers (good or very good) was almost universal (96.1%) although nearly half of the patients (53.6%) acknowledged that they had to wait more than 30 min before receiving service. The long waiting time was associated with CAM use (*p* = 0.015). Two–thirds of the patients thought that the cost of treatment provided at healthcare facilities was affordable (65.0%). The majority of the patients declared that they were globally satisfied with the quality of healthcare (86.8%).

**Table 7:** Healthcare system factors

	Overall *n* = 280 (% or SD)	CAM users *n* = 73 (% or SD)	CAM non-users *n* = 207 (% or SD)	*p*
Availability of HCF				
Not available	34 (12.1)	7 (9.6)	27 (13.0)	0.437
Available	246 (87.9)	66 (90.4)	180(87.0)	
Waiting time				
Very long/long	150 (53.6)	48 (65.8)	102(49.3)	0.015^a^
Very short/short	130 (46.4)	25 (34.2)	105(50.7)	
Relationship				
Somewhat good/bad	11 (3.9)	3 (4.1)	8 (3.9)	0.581
Very good/good	269 (96.1)	70 (95.9)	199 (96.1)	
Affordability				
Not affordable	98 (35.0)	26 (35.6)	72 (34.8)	0.898
Affordable	182 (65.0)	47 (64.4)	135(65.2)	
Global satisfaction				
No	37 (13.2)	11 (15.1)	26 (12.6)	0.586
Yes	243 (86.8)	62 (84.9)	181(87.4)	

^a^Statistically significant

### Determinants of CAM use

Table 8 summarizes the determinants of CAM use with bivariate and multivariate analysis. Bivariate analysis shows that the lack of knowledge of treatment benefits by patients (OR = 2.3; 95%CI: 1.1-4.8), the experiencing of side effect from medication (OR = 3.2; 95% CI: 1.8─5.9) and the long waiting time (OR = 2.0; 95% CI: 1.1─3.6) were associated with CAM use. The misperception of HTN curability (OR = 1.8; 95%CI: 0.97-3.3) has been also included in the multivariate analysis because its *p* value (0.045). Multivariate analysis identified a misperception by patients of HTN curability (OR = 2.1; 95%CI: 1.1-3.7) and experiencing of side effects from medication (OR = 2.9; 95%CI: 1.7-5.1) as predictors of CAM use.

**Table 8:** Bivariate and multivariate analysis determinants of CAM use

Factors	CAM use					
	Bivariate analysis			Multivariate analysis (LR)		
	Crude OR	(95% CI)	*p*	Adjusted OR	(95% CI)	*p*
Knowledge of treatment benefits						
Have cited less than 3	2.3	(1.1–4.8)	0.022^a^			
Have cited a least 3	1	-	-	-	-	-
Perception of HTN curability						
HTN is curable	1.8	(0.97–3.3)	0.045^a^	2.1	(1.1–3.7)	0.019^a^
HTN is incurable	1	-	-	-	-	-
Experiencing side effects						
Yes	3.2	(1.8–5.9)	0.000^a^	2.9	(1.7 – 5.1)	0.000^a^
No	1	-	-	-	-	-
Waiting time						
Very long/long	2.0	(1,1–3.6)	0,015^a^			
Very short/short	1	-	-	-	-	-

^a^Statistically significant

### Patient’s characteristics and perception of HTN curability

Table 9 shows that the duration of HTN (*p* = 0.022) and religious affiliation (0.006) were statistically associated with the misperception of HTN curability.

**Table 9:** The perception of HTN curability and patient’s characteristics

		Perception of HTN curability		
	Overall (*n* = 280)	HTN is curable (*n* = 172)	HTN is incurable (*n* = 108)	*p*
Mean age, years (SD)	60.1(10.6)	60.2(11.0)	60.0(9.8)	0.879
Sex				
Male	88 (31.4)	54 (61.4)	34 (38.6)	0.988
Female	192 (68.6)	118 (61.5)	74 (38.5)	
Educational level				
Primary	85 (30.6)	54 (63.5)	31 (36.5)	0.691
Secondary	142 (51.1)	85 (59.0)	59 (41.0)	
Post-secondary	51 (18.3)	33 (64.7)	18 (35.3)	
Religious affiliation				
Catholic	121 (43.2)	65 (53.7)	56 (46.3)	0.006^a^
Pentecostal	57 (20.4)	46 (80.7)	11(19.3)	
Protestant	48 (17.1)	29(60.4)	19(39.6)	
Kimbanguist	22 (7.9)	16 (72.7)	6 (27.3)	
Jehovah’s witnesses	13(4.6)	5 (38.5)	8(61.5)	
Others	19 (6.8)	11 (57.9)	8(42.1)	
Duration of HTN				
≥ 5 years	89 (31.8)	46 (51.7)	43 (48.3)	0.022^a^
< 5 years	191(68.2)	126 (66.0)	65(34.0)	

^a^Statistically significant

## Discussion

The study found that more than a quarter of patients used CAM (26.1%); mainly the biologically based therapies and the mind-body interventions.

The misperception of HTN curability and experience of medication side effects were independent determinants of CAM use. Religious affiliation and HTN duration were associated with misperception of HTN curability.

The present study confirmed our hypothesis asserted in a study conducted previously that a sizeable proportion of patients with hypertension in Kinshasa use alternative care [14].The proportion of the use of CAM found in this study 26.1% (95% CI 20.7%-31.8%) is similar to that found by previous studies conducted in sub-Saharan Africa (SSA) [20, 21].

Alternative treatments used by the majority of respondents included medicinal plants by self-medication or prescribed by herbalists, followed by prayer. These findings are similar to those found in previous studies [15, 22]. This can be explained in the current context of the city of Kinshasa where so many commercial traditional healers promote the values of their plants, pretending that they can cure even incurable diseases such HTN, and DM. Amira and Okubadejo in Nigeria observed that powerful advertisements placed by alternative medicine practitioners encouraged patients to use as a remedy for all diseases, a panacea [23]. Thus, they advocate healing without regard to the observance of certain dietary measures that are described as an important factor affecting the quality of life of patients.

The health expectations of patients are linked to adherence to conventional treatment and to use of CAM. The CAM practitioners interviewed during a qualitative study carried out in Nigeria declared that the main reason patients consulted them was the belief that CAM practitioners could cure HTN at a more reasonable cost than the HCF [23]. The perceived effectiveness and low cost were the main reasons for the use of CAM cited in this study. The study found that patients who believed that hypertension is curable were more likely to use CAM.

Literature indicates that in developing countries, the population has a strong belief in the efficacy of natural and local resources in treating diseases. These resources are described by people as having no side effects unlike modern medicine [17]. This study also found that the presence of side effects of medications was independently associated with the use of alternative care; this result is consistent with earlier studies [17, 24–26].

Healthy behavior models increasingly describe the impact of social determinants such as spirituality and religion on health and treatment adherence [27]. Spirituality and religion can lead to erroneous beliefs that can lead to the denial of the disease which is a determinant of non-adherence [28]. Spiritual and religious beliefs can therefore have a significant effect on health behavior. The study found that more than two thirds of hypertensive patients thought they can cure their disease and this misperception was a factor in the use of alternative medicine. The misperception of HTN curability was associated with religious affiliation, majority of patients of Pentecostal churches declared that HTN is curable comparatively to others religious affiliation. For more than a decade, there has been an influx of Pentecostal churches in Kinshasa that have brought a new current of thought of all fields of life of the populace. These churches are authors of miracle cures and advocate the supremacy of God who heals; to accept an incurable chronic disease would be to ignore the power of God. Religious leaders are important authority figures for the population of Kinshasa and the former could constitute an important target for communication on behavioral change.

The study did not find any association between NAM and CAM use. This can be explained by the tool used for measuring NAM -the MMAS, comprising of four questions, which probably failed to detect that some people can refuse to take their medicine because they believe that HTN can be cured by God.

The study was conducted in hospitals and thus probably underestimated the frequency of the use of alternative care. Furthermore NAM was measured using a self-reported questionnaire. Nevertheless, this study is one of the first to have described this problem in the DRC and the results found are consistent with those described in the literature.

## Conclusion

The use of CAM for hypertensive patients attending the primary healthcare facilities in Kinshasa is a major problem; antihypertensives with fewer side effects must be emphasized and religious leaders must become involved in the communication and advocation of behavioral change in order to improve the quality of life of hypertensive patients.

## Additional file

Additional file 1: Dataset. This file contains data supporting the results of this study. (DTA 405 kb)

## Abbreviations

BP: Blood pressure
CAM: Complementary and Alternative Medicine
CKD: Chronic kidney disease
CVD: Cardiovascular diseases
CVRF: Cardiovascular risk factors
DM: Diabetes Mellitus
DRC: Democratic Republic of Congo
HCF: Healthcare Facility
HS: Health System
HTN: Hypertension
KPHC: Kinshasa Primary Health-care
MMAS: Morisky Medication Adherence Scale
NAM: Non-adherence to antihypertensive medication
NCDs: Non communicable diseases
NHI: National Institute of Health
OR: Odds Ratio
SSA: Sub-Saharan Africa
WHO: World Health Organization
